# The Current Era of Endovascular Aortic Interventions and What the Future Holds

**DOI:** 10.3390/jcm11195900

**Published:** 2022-10-06

**Authors:** Martin Teraa, Constantijn E. V. B. Hazenberg

**Affiliations:** Department of Vascular Surgery, University Medical Center Utrecht, 3584 CX Utrecht, The Netherlands

Today, more than 30 years after the first endovascular aneurysm repair (EVAR) by Juan Parodi and Julio Palmaz [1], endovascular aortic interventions have become the preferred treatment modality for a wide range of aortic pathologies. We have long passed the era in which endovascular aortic interventions were confined to simple infrarenal abdominal aortic aneurysms (AAA); nowadays, complex aortic pathologies, such as juxtarenal AAA, extensive thoraco-abdominal aneurysms, AAAs with challenging neck or access anatomies, aortic dissections, and even pathology of the aortic arch, can be treated via a complete or hybrid endovascular approach. Despite important advantages, endovascular (aortic) interventions, compared with traditional vascular surgery, also bring new challenges and demands for further innovation to achieve the best outcome for the patient and the treating physician. This applies for the complete course of the treatment of patients with aortic disease, i.e., the pre-operative, peri-operative, and post-operative phases.

## 1. Pre-Operative Phase and Planning

Patient selection has changed, and the indications for endovascular aortic interventions have broadened over the past decade, which goes hand in hand with more complex pathologies being treated with minimally invasive procedures. For example, patients unfit for open repair of an arch aneurysm can be treated with a complete endovascular or hybrid approach. Accurate knowledge of all (endovascular) options and their corresponding advantages and drawbacks is essential. Consequently, the question has been raised as to whether these complex interventions should be performed in all centers equipped with modern hybrid operation rooms (ORs) or only in high-volume centers. In line with what has been repeatedly shown for major surgical procedures, mortality and major complications after complex endovascular interventions, such as fenestrated (FEVAR) and branched EVAR (BEVAR), are substantially higher (up to 4 times higher) in low-volume than high-volume centers [2,3,4]. These results underline the importance of the centralization of these complex interventions, which is likely to become even more important in the near future.

Intervention-related decision making based on pre-operative planning and stent graft sizing and selection has evolved enormously in recent decades, these factors directly influence initial technical success, the durability of aortic interventions, as well as the risk of complications [5,6]. Dynamic properties of the aorta during the cardiac cycle and dimensional changes due to hemodynamic shifts make sizing in aortic interventions challenging, especially in acute aortic syndromes such as blunt traumatic aortic injury (BTAI). To optimize outcomes, methods to guide treatment planning are mandatory. For instance, it has been shown that the real-time assessment of aortic diameters using intravascular ultrasound (IVUS) to support stent graft selection in acute aortic syndromes improves post-operative outcomes. Other promising technologies that are increasingly used in different medical fields, such as machine learning and artificial intelligence (AI), have still to be proven in the routine management of patients with aortic pathology. Although, its role in fully automated volume segmentation [7] and treatment planning [8] has been shown in infrarenal AAA, its value in pre-operative planning and stent graft selection and sizing is yet to be established. Furthermore, patient selection and screening, per-operative guidance, and individual patient’s post-operative follow-up planning could benefit from AI methods. Therefore, AI or deep learning algorithms will become indispensable tools in the future management of patients with aortic disease.

## 2. Per-Procedural Phase

Per-operative image guidance is an essential element in the chain of endovascular interventions and especially complex aortic procedures. Image guidance has evolved enormously in recent years and, consequently, endovascular navigation during complex endovascular aorta interventions has improved. However, this requires fluoroscopy. Fluoroscopically guided endovascular interventions have some important limitations: (1) the acquired images are a two-dimensional (2D) conversion of three-dimensional (3D) structures and movements, (2) images are projected only in gray scale, and importantly (3) it requires radiation exposure. Increasing attention has been paid to these important drawbacks of fluoroscopy and, importantly, the awareness of occupational radiation exposure during these interventions has increased. This increased awareness is reflected by the 2023 clinical practice guidelines on radiation safety by the European Society for Vascular Surgery (ESVS) [9], which give firm recommendations and expose knowledge gaps regarding radiation safety during endovascular (aortic) interventions. 

A state-of-the-art hybrid OR has options to perform image fusion, which enables merging pre- or per-operative imaging, such as CTA or MRA, with the real-life images on the hybrid OR. Image fusion enables navigation within a 3D roadmap and easier and more accurate navigation. It has been shown that the use of image fusion reduces contrast volume, fluoroscopy and procedure time in complex EVAR, but influence on radiation dose has not been substantial [10]. In order to reduce or even banish radiation from the OR, radiation-free techniques must be investigated and developed to pursue radiation-free endovascular surgery. Promising techniques are IVUS, electromagnetic tracking (EM) robotic navigation, and Fiber Optic RealShape (FORS).

Fully IVUS-assisted EVAR has been shown to be feasible in twenty-seven cases and to significantly reduce the amount of radiation exposure and contrast volume during EVAR procedures [11,12]. Although IVUS in itself is not novel, its application in aortic interventions is still very limited, but it could be one of the methods to reduce radiation exposure during aortic interventions significantly.

EM-tracking systems consist of a low-magnetic-field generator and EM position coils integrated within the tip of the used catheter or guidewire. Information about the EM field within the EM coils at the tip of the devices is analyzed in a control box that converts this information into a 3D position of the coil. In combination with navigation software, the system can visualize the 3D position and orientation of the devices relative to the anatomy, segmented from a preoperative CTA. Most articles describing EM tracking have reported results of in vitro and animal studies [13]; however, its feasibility and potential in endovascular aortic surgery have been shown in small clinical studies [14,15,16]. Larger studies have yet to confirm the additional value and radiation-reducing capacity of EM tracking during complex aortic procedures.

Finally, an important and promising innovation that should ease 3D navigation and reduce radiation exposure in endovascular interventions is FORS technology [17]. FORS technology makes use of special designed guidewires and catheters with an integrated optical fiber. Positional changes in the devices alter the optical signal and the FORS software visualizes the actual position of the devices in real time. FORS technology can be combined with image fusion. Important advantages of FORS include a better appreciation of 3D movements, visualization in bright colors, the option of simultaneous biplane view, and real-time navigation without the use of fluoroscopy. FORS technology has been successfully adopted in complex endovascular aortic repair programs in selected high-volume aortic centers, and initial results show encouraging success rates and high potential for radiation reduction [18].

Although most of the abovementioned methods are still not routinely available in daily practice, they will help us to shape the radiation-free hybrid ORs and angiosuites of the future. In addition to imaging and radiation-reducing innovations, the development of a new generation of endografts enables the treatment of wider and more complex aortic pathologies (e.g., hostile necks or atherosclerotic iliac access). These innovations require tight collaboration between vascular surgeons and broad groups of specialists, such as technicians, IT specialists, basic and clinical scientists, and industry representatives. This will fuel these innovations and speed up the translation of these novel techniques towards our ORs.

## 3. Post-Operative Phase and Follow-Up

Compared with traditional aortic reconstructions, endovascular aortic interventions also differ in post-operative follow-up. For instance, endoleaks are the Achilles heel of EVAR, and have varying consequences depending on the type and presence of aneurysm sac expansion. Especially, the role and importance of type 2 endoleaks remain a matter of debate, and it would be significant if we could identify clinically relevant endoleaks, as less than 1% of patients with a type 2 endoleak will eventually develop a ruptured aneurysm. It has been shown that machine learning algorithms are able to reliably predict those endoleaks related to significant aneurysm sac expansion [19], as these aneurysms are more prone to rupture than the stable ones. Thus, this could help with the selection of patients in whom the type 2 endoleak should be treated. Furthermore, similar techniques have been able to predict reinterventions after thoracic endovascular aortic repair (TEVAR) for type B aortic dissection [20]. Methods to inform tailor-made follow-up and guide reinterventions will further improve the long-term results of endovascular aortic interventions, prevent unnecessary imaging and reinterventions, and ultimately reduce costs.

## 4. Conclusions

In conclusion, this Special Issue addresses important aspects of modern endovascular aortic interventions, some of which have been discussed in this Editorial, and casts a view on future developments in this fast-moving field. We sincerely hope that this Special Issue will help to increase insight in endovascular aortic interventions and fuel the next steps in innovation and personalized care. This will ultimately help to improve outcomes for both the patients, suffering from serious and often life-threatening aortic pathologies, and for us, as vascular surgeons and interventional radiologists, by attempting to reduce and finally banish radiation exposure and achieve durable results.

We would like to thank all reviewers for their insightful comments and help to further improve the manuscripts included in this Special Issue and the *JCM* team for their support. Additionally, foremost, we heartily thank the authors for their valuable and high-quality contributions which have shaped this Special Issue and will help to shape the future of endovascular aortic surgery.
